# Association of Recent Violence Encounters With Suicidal Ideation Among Adolescents With Depression

**DOI:** 10.1001/jamanetworkopen.2023.1190

**Published:** 2023-03-02

**Authors:** Jing Wang, Shannon Harrer, Marissa L. Zwald, Ruth W. Leemis, Kristin M. Holland, Deborah M. Stone, Kathleen McDavid Harrison, Elizabeth A. Swedo

**Affiliations:** 1 Division of Injury Prevention, National Center for Injury Prevention and Control, US Centers for Disease Control and Prevention, Atlanta, Georgia; 2 Palantir Technologies, Cambridge, Massachusetts; 3 Division of Violence Prevention, National Center for Injury Prevention and Control, US Centers for Disease Control and Prevention, Atlanta, Georgia

## Abstract

**Importance:**

Suicide prevention is an important component of depression management. Knowledge about depressed adolescents with increased risk for suicide can inform suicide prevention efforts.

**Objective:**

To describe the risk of documented suicidal ideation within a year following a diagnosis of depression and to examine how the risk of documented suicidal ideation differed by recent violence encounter status among adolescents with new depression diagnoses.

**Design, Setting, and Participants:**

Retrospective cohort study in clinical settings including outpatient facilities, emergency departments, and hospitals. Using IBM’s Explorys database containing electronic health records from 26 US health care networks, this study observed a cohort of adolescents with new depression diagnoses from 2017 to 2018 for up to 1 year. Data were analyzed from July 2020 to July 2021.

**Exposures:**

Recent violence encounter was defined by a diagnosis of child maltreatment (physical, sexual, or psychological abuse or neglect) or physical assault within 1 year before depression diagnosis.

**Main Outcomes and Measures:**

The main outcome was diagnosis of suicidal ideation within 1 year following depression diagnosis. Multivariable adjusted risk ratios of suicidal ideation were calculated for overall recent violence encounters and for individual forms of violence.

**Results:**

Among a total of 24 047 adolescents with depression, 16 106 (67.0%) were female and 13 437 (55.9%) were White. A total of 378 had experienceda violence (hereafter, encounter group) and 23 669 had not (hereafter, nonencounter group). Following the diagnosis of depression, 104 adolescents with any past-year violence encounter (27.5%) documented suicidal ideation within 1 year. In contrast, 3185 adolescents in the nonencounter group (13.5%) experienced thoughts of suicide following the diagnosis of depression. In multivariable analyses, those with any violence encounter had 1.7 times (95% CI 1.4–2.0) higher risk of documented suicidal ideation compared with those in the nonencounter group (*P* < .001). Among different forms of violence, sexual abuse (risk ratio, 2.1; 95% CI, 1.6–2.8) and physical assault (risk ratio, 1.7; 95% CI, 1.3–2.2) were associated with significantly increased risk of suicidal ideation.

**Conclusions and Relevance:**

Among adolescents with depression, persons who experienced past-year violence encounters showed a higher rate of suicidal ideation than those who had not. These findings highlight the importance of identifying and accounting for past violence encounters when treating adolescents with depression to reduce risk of suicide. Public health approaches to prevent violence may help to avert morbidity associated with depression and suicidal ideation.

## Key Points

**Question:**

Among adolescents with depression diagnoses, are recent violence encounters associated with elevated risk for suicidal ideation?

**Findings:**

In this cohort study of 24 047 adolescents with new depression diagnoses, more than a quarter (27.5%) of adolescents who experienced past-year violence encounters documented suicidal ideation within 1 year. Recent violence encounters were associated with 1.7 times higher risk for documented suicidal ideation among adolescents with depression.

**Meaning:**

These findings suggest that to prevent suicide, it may be important to consider recent violence encounters when managing adolescent depression.

## Teaser

This cohort study evaluates the association of recent violence encounters with suicidal ideation among adolescents with depression.

## Introduction

In 2019, as many as 1 in 6 adolescents in the US reported experiencing major depressive disorder during the past year.^1^ Depression is the most common mental health condition precipitating suicide,^2^ making it a crucial target for suicide prevention among adolescents.^3^ Fortunately, most adolescents with depression do not have suicidal ideation or behaviors.^4^ Improved knowledge on distinct groups at increased risk for suicide among adolescents with depression could facilitate effective suicide prevention.

Violence encounters are a well-recognized risk factor for suicidal ideation or behavior among the general youth population.^5,6,7,8^ The link between violence and suicide is theorized to be mediated, in part, through depression^6^ but has seldom been studied among adolescents with depression. It is unclear whether violence encounters elevate risk for suicidal ideation or behavior among those with depression. Fergusson et al^4^ showed that sexual abuse during childhood increased the risk for suicidal ideation or behavior among youth with depression. Their findings underscored that violence encounters as an adverse childhood experience have deleterious effects on mental health and well-being^9,10^ and may increase vulnerability to suicide in later years. Moreover, violence encounters can have immediate psychological and behavioral sequelae and may affect suicide risk in the short term.^11^ Recent exposure to adversity has been found to have particularly strong negative effects on adolescents’ physical health when compared with adverse experiences during younger childhood.^12^ As such, particular emphasis should be given to studying recent violence encounters and suicidal behavior. Foodward et al^13^ reported that recent assault was associated with suicide attempts among adolescents with depression in a primary care setting; however, this cross-sectional study could not define the chronological sequence of being assaulted and suicide attempt. To our knowledge, there have been no longitudinal studies examining the impact of recent violence encounters and the risk of suicidal ideation or behavior among adolescents with depression.

Using the IBM Explorys Electronic Health Record database, we conducted a retrospective cohort study of adolescents with a new diagnosis of depression during 2017 to 2018 with the goals to: (1) describe short-term risk of documented suicidal ideation at the time of depression diagnosis; and (2) examine the association of recent violence encounters (ie, child maltreatment and physical assault) with short-term risk of suicidal ideation. We focused on the time when depression was diagnosed, as this clinical encounter represents both a high-risk time for suicidal ideation or behavior^14^ and a window of opportunity for suicide risk assessment and management among adolescents with depression.

## Methods

### Data Source

Explorys data from 2016 to 2019 were analyzed in this retrospective cohort study. Explorys^15^ is a longitudinal patient-level electronic health record database covering 15% of the US population and spanning 50 states. It integrates clinical medical records and claims data for more than 64 million patients in 26 leading health care networks covering 920 000 practitioners. We used *International Statistical Classification of Diseases, Tenth Revision, Clinical Modification (ICD-10-CM)* for all diagnoses, Systematized Nomenclature of Medicine codes, and Logical Observation Identifiers Names and Codes to define the clinical conditions as presented in eTable 1 in Supplement 1. Because this study was a secondary analysis of deidentified data, the US Centers for Disease Control and Prevention determined that institutional review board review and the need for informed consent were exempt. This report follows the Strengthening the Reporting of Observational Studies in Epidemiology (STROBE) reporting guideline for cohort studies.^16^

### Study Population

Two nonoverlapping cohorts of adolescents aged 10 to 19 years were created to increase sample size, namely the 2017 cohort and the 2018 cohort. The study period for the 2017 cohort took place from January 1, 2016, through December 31, 2018. This cohort comprised adolescents with a new *ICD-10 CM* diagnosis of depression in 2017, defined as patients without any past-year documented diagnosis of depression or antidepressant prescriptions. The date of diagnosis of depression is referred to as the index date. The study population was limited to adolescents who continuously enrolled in Explorys during the study period, defined as having at least 1 encounter for any reason in each year of 2016 to 2018. Following a similar process, a 2018 cohort comprised adolescents with a new diagnosis of depression in 2018 who continuously enrolled during January 1, 2017, through December 31, 2019 (eFigure in Supplement 1).

### Exposure

Our exposure was defined by *ICD-10-CM* diagnosis of violence encounter occurring within 1 year before the index date (this group of patients will be referred to as the encounter group hereafter). Specifically, we included child maltreatment (physical, sexual, or emotional abuse or neglect by a parent or other caregiver) and physical assault (injuries inflicted by someone other than the parent or caregiver with the intent to injure or kill). We studied exposure to any violence as well as exposure to each form of violence. If more than 1 act of violence was documented, we used the most recent act recorded before the index date. Patients without a diagnosis of past-year violence encounter (hereafter referred to as the nonencounter group) served as the comparison group.

### Outcome

This study focused on suicidal ideation because suicide attempts and death by suicide were suppressed in the Explorys data set. However, suicidal ideation is an important factor associated with risk for suicide and suicide attempts. About one-third of adolescents experiencing suicidal ideation will attempt suicide before adulthood. Among individuals who attempted suicide, most (86%) occurred within a year of having suicidal ideation,^17^ highlighting the importance of examining suicide ideation. The study outcome was defined as an *ICD-10-CM* diagnosis of suicidal ideation within 1 year of the index depression diagnosis, which measured short-term suicidal risk that can be especially pertinent to planning for patient care during their clinical encounters.

#### Covariates

We classified patients’ characteristics at the time of depression diagnosis into the following categories: age group (10–14 years, 15–19 years), sex (male or female according to biological sex), race and ethnicity (Black, Hispanic, White, or other/missing [other race and ethnicity includes American Indian or Alaskan Native, Asian, Native Hawaiian or Other Pacific Islander, or multiracial]), and insurance type (public, private, or other/missing). Because either race or ethnicity, but not both, are often listed in Explorys, when Hispanic ethnicity was specified, it took precedence over race. ‘Missing’ was combined with ‘other’ for the race and ethnicity and insurance variables in statistical modeling due to small numbers in some violence encounter categories. Race and ethnicity were analyzed as a covariate because it may be a confounder for the association of violence encounters and suicidal ideation.

Preexisting substance use was defined as documentation of alcohol use, tobacco use, or other substance use at any time before the index date. Alcohol use and smoking status were identified according to patient self-reported behavior or clinician-based *ICD-10-CM* diagnosis. *ICD-10-CM* diagnoses were used to identify other substance use. As substance use may be a sequela of violence encounters^11^—and thus is regarded as a mediator—substance use documented after the most recent violence encounter was not included as a covariate for the encounter group to avoid overadjusting for the effects of substance use.

Mental illness was defined as any diagnosis of autism spectrum disorder, attention-deficit/hyperactivity disorder, conduct disorder, or schizophrenia before the index date and was treated as confounders associated with violence encounters^18^ and suicidal ideation.^19,20^ Anxiety, often a sequela of violence encounters,^11^ was considered a mediator and therefore was not adjusted for in the model. Detailed algorithms to define all conditions are presented in eTable 1 in Supplement 1. The specified timings relative to the index date in the definitions of each condition are summarized in eTable 2 in Supplement 1.

### Statistical Analyses

#### Main Analysis

From an initial population of 482 715 patients with a new diagnosis of depression, we excluded patients who were ineligible to participate because they did not enroll for the entire 3-year study period (238 143 patients) or were beyond the age range of 10 to 19 years (219 494 patients). To focus on adolescents with depression as the study population, patients who had a diagnosis of suicidal ideation before the index date were excluded because they might not have depression when having suicidal ideation (795 patients). Lastly, to study recent violence encounters, patients whose latest violence encounter was more than 1 year before the index date were excluded (236 patients).

Statistical analyses were conducted in R version 3.5.1 (R Project for Statistical Computing). We computed adjusted risk ratios (RR) of suicidal ideation for those with violence encounters compared with those with no encounters according to multivariable Poisson regression using robust standard errors.^21^ In separate models, we obtained RRs for overall violence encounters, as well as for each form of violence encounter relative to no encounters. We did not estimate RR for child neglect due to the small sample size (6 participants). Age, sex, race and ethnicity, and insurance type, in addition to history of substance use and mental illness were used for adjustment in the model. A 2-sided *P* < .05 was considered significant. Data were analyzed from July 2020 to July 2021.

#### Validation Analyses

Given that this study was based on diagnosed cases, we conducted post hoc validation analyses to evaluate potential sources of bias. Regarding potential detection bias on the outcome, we assessed whether the encounter group were more likely to be screened for suicidal ideation than the nonencounter group. We compared the percentage of adolescents receiving the 9-item Patient Health Questionnaire (PHQ-9)^22^ assessment in the month before, or 3 months after, the index date by violence encounter status. Furthermore, children of racial and ethnic minorities or with nonprivate insurance are frequently overevaluated for child abuse,^23^ although mainly on physical abuse among young children. Adolescents with these socioeconomic characteristics are disproportionally affected by various early-life adversities that may increase risk for suicide.^24,25^ Their being overrepresented in the encounter group due to more detection may mislead the results. Thus, we compared the distributions of race and ethnicity and insurance type among adolescents with violence encounters as well as among adolescents with depression in our study vs in national population-based surveys.^26,27,28,29^ Lastly, to ensure that our findings were not affected by antidepressant treatment that may be associated with the potential risk of increased suicidal ideation,^30^ we redefined outcome as suicidal ideation on the same day instead of within 1 year of depression diagnosis when the patients had not been treated with antidepressants.

## Results

**Figure 1.**
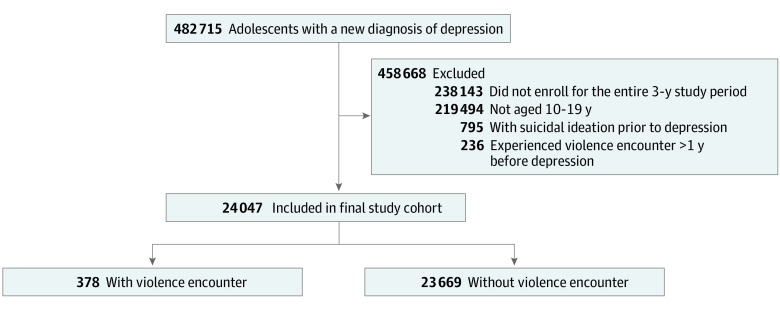
Study Cohort Accrual Flowchart Study period was 2016 to 2018 for the 2017 cohort and 2017 to 2019 for the 2018 cohort. The violence encounter group was composed of adolescents with a diagnosis of any violence encounter (child maltreatment–physical, sexual, or emotional abuse or neglect) and physical assault within 1 year before the index date. Otherwise, the adolescent was classified as nonencounter group.

A total of 24 047 adolescents (16 106 [67.0%] female and 13 437 [55.9%] White) had a new diagnosis of depression during 2017 and 2018, among whom 1.6% (378 adolescents) had a violence encounter within the past year (Figure 1). Assault (161 participants [42.6%]) was the most common form of violence encounter, followed by sexual abuse (93 participants [24.6%]), psychological abuse (72 participants [19.0%]), physical abuse (33 participants [8.7%]), and neglect or other violence (19 participants [5.0%]). Compared with the nonencounter group, the encounter group was more likely to be younger (age 10–14 years), female, Black or Hispanic, and covered by public insurance. In addition, the encounter group had a higher percentage of previous substance use and mental illness compared with the nonencounter group (Table 1).

**Table 1.** Demographic Characteristics for the Study Population by Violence Encounter Status^a^

Characteristic	No. (%)		*P* **value**^b^
	Nonencounter group (N = 23 669)	Encounter group (N = 378)	
Suicidal ideation	3185 (13.5)	104 (27.5)	<.001
Age group, y^c^			
10-14	6972 (29.5)	134 (35.4)	.01
15-19	16 697 (70.5)	244 (64.6)	
Sex			
Female	15 826 (66.9)	280 (74.1)	.008
Male	7760 (32.8)	98 (25.9)	
Missing	83 (0.4)	0	
Race and ethnicity			
Black	2273 (9.6)	81 (21.4)	<.001
Hispanic	1648 (7.0)	44 (11.6)	
White	13 242 (55.9)	195 (51.6)	
Other^d^^,^^e^	6506 (27.5)	58 (15.3)	
Type of health insurance			
Private plan	12 811 (54.1)	128 (33.9)	<.001
Public plan	5372 (22.7)	144 (38.1)	
Other^e^	5486 (23.2)	106 (28.0)	
Substance use^c^	2378 (10.0)	71 (18.8)	<.001
Mental illness^c^	5203 (22.0)	100 (26.5)	.04
PHQ-9 assessment^c^	2669 (11.3)	45 (11.9)	.76

Abbreviation: PHQ-9, Patient Health Questionnaire assessment.

^a^ Data source: IBM Explorys Electronic Health Record Database.

^b^ The distributions in demographic characteristics were compared between the encounter group and nonencounter group according to χ^2^ test.

^c^ Missing data are not reported for these variables. Because the study population was specified for adolescents aged 10-19 years, there was no missing value for age group. For substance use and mental illness that were identified by *ICD-10-CM* diagnosis codes, the records in absence of the specified codes were defined as “no.” Records without indicating “PHQ-9 assessment” were treated as “no.”

^d^ Other race and ethnicity includes American Indian or Alaskan Native, Asian, Native Hawaiian or Other Pacific Islander, or multiracial.

^e^ The category of “missing” was combined with “other” for the race and ethnicity and health insurance variables in statistical modeling due to small numbers in some violence encounter categories.

**Figure 2.**
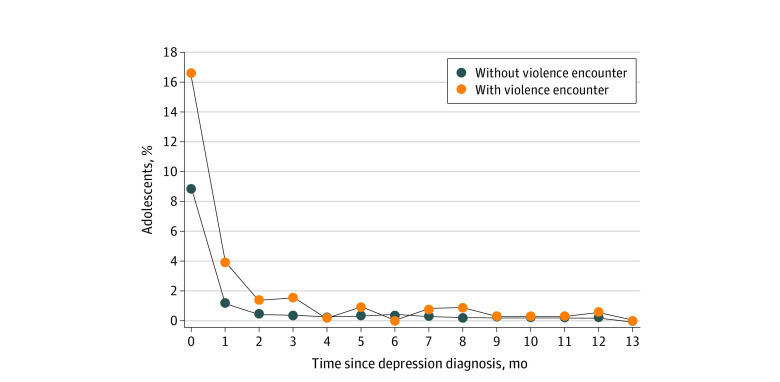
The Timing of Initial Documented Suicidal Ideation Following Depression Diagnosis Data source: IBM Explorys Electronic Health Record Database. The percentage was calculated as number of adolescents (aged 10-19 years) with documented suicidal ideation during each time frame divided by the total number of adolescents for the encounter group and the nonencounter group, respectively. The months since depression diagnosis date was calculated using the following values: the month value of 0 indicates documented suicidal ideation and depression on the same day; the value of 1 or greater indicates documented suicidal ideation within the number of months after depression diagnosis.

Overall, 13.7% of adolescents (3289 adolescents) had a diagnosis of suicidal ideation in the year following depression diagnosis. Among the encounters group, 27.5% (104 adolescents) had documented suicidal ideation, in contrast to 13.5% (3185 adolescents) in the nonencounter group (χ^2^_1_ = 62.3; *P* < .001). Regarding timing of suicidal ideation, the initial documented suicidal ideation was most commonly identified simultaneously at the time of depression diagnosis (16.7% and 8.9% of patients from the violence encounter group and nonencounter group, respectively), accounting for about 61% and 66% of total cases with suicidal ideation in each group. (Figure 2).

After adjusting for covariates, among adolescents with depression, the encounter group had 1.7 (95% CI, 1.4-2.0) times the risk of suicidal ideation compared with the nonencounter group (Table 2). Regarding specific forms of violence encounters, recent sexual abuse (RR, 2.1; 95% CI, 1.6-2.8) or assault (RR, 1.7; 95%, CI 1.3-2.2) were found to be significantly associated with increased risk of suicidal ideation. Adolescents with a history of substance use had 1.7 times the risk (95% CI, 1.6-1.9) of suicidal ideation compared with those without substance use. In addition, younger age (10-14 years), people of Black race or Hispanic ethnicity, and people with public insurance had a higher risk of suicidal ideation. Neither history of other mental illness nor sex resulted in significant associations with suicidal ideation.

**Table 2.** Adjusted Risk Ratio for Suicidal Ideation Associated With Violence Encounters Among Adolescents With Depression^a^^,^^b^

Characteristic	Adjusted risk ratio (95% CI)	*P* value
Violence encounter^c^		
No	1 [Reference]	
Yes	1.7 (1.4-2.0)	<.001
Violence form^c^		
No encounters	1 [Reference]	
Physical abuse	1.4 (0.7-2.7)	.36
Sexual abuse	2.1 (1.6-2.8)	<.001
Psychological abuse	1.4 (0.9-2.2)	.17
Assault	1.7 (1.3-2.2)	<.001
Age, y		
10-14	1 [Reference]	
15-19	0.8 (0.8-0.9)	<.001
Race and ethnicity		
White	1 [Reference]	
Black	1.1 (1.0-1.2)	.02
Hispanic	1.2 (1.1-1.3)	.002
Other^d^	0.6 (0.5-0.6)	<.001
Insurance		
Private	1 [Reference]	
Public	1.4 (1.3-1.5)	<.001
Other	0.9 (0.8-1.0)	.01
Substance use		
No	1 [Reference]	
Yes	1.7 (1.6-1.9)	<.001

^a^ Data source: IBM Explorys Electronic Health Record Database.

^b^ Sex and mental illness were not kept in the final multivariable model as they became nonsignificant after adjusting for other variables and neither significantly changed the coefficient for violence encounter status.

^c^ In separate models, we obtained risk ratios for overall violence encounters as well as for each form of violence encounter (physical abuse, sexual abuse, psychological abuse, assault) relative to no encounters and adjusting for covariates. Risk ratio for neglect was not estimated due to small sample size (6 participants).

^d^ Other race and ethnicity includes American Indian or Alaskan Native, Asian, Native Hawaiian or Other Pacific Islander, or multiracial.

For validation analyses, screening rates using the PHQ-9 were similar between adolescents with violence encounters and those without encounters (Table 1), suggesting a detection bias on suicidal ideation was not present. Among encounters, the percentage of racial and ethnic minority groups was slightly lower in our study than in national population-based surveys (eTable 3 in Supplement 1). Among adolescents with depression, the percentages of racial and ethnic minority groups or nonprivate insured were comparable in our study as in a national survey, which do not suggest overrepresentation of children of racial and ethnic minorities or with nonprivate insurance in our sample (eTable 4 in Supplement 1). When we redefined outcome as suicidal ideation on the same day of the index date to eliminate potential impact from antidepressant treatment, our conclusions remained unchanged.

## Discussion

To our knowledge, this study is the first longitudinal study to comprehensively examine the association of recent violence encounters and suicidal ideation among adolescents with depression. We observed a high rate (27.5%) of documented suicidal ideation within a year of depression diagnosis among adolescents in the violence encounter group. An estimated 1.4 million children and youth made violence-related medical visits per year, of whom two thirds are adolescents.^31^ Our findings underscore the importance of addressing violence exposure when adolescents make contact with the health care system to curtail mental illness trajectories and suicide risk.^32^ Unfortunately, violence exposure frequently goes undetected in health care settings, implying missing opportunities to provide needed services or therapeutic interventions.^33^ Currently, there is no consensus on when or how to conduct clinical screening for traumatic experiences, including violence encounters.^34,35^ Further efforts are warranted to develop best practices in identifying violence encounters to guide appropriate clinical care.

Literature indicates that people with depression who experience interpersonal violence may differ from those who do not experience interpersonal violence regarding clinical courses of depression, such as earlier age of onset of disease, more episodes, more comorbidities, and delayed recovery of depression.^36,37,38^ History of violence exposure may also moderate individuals’ responses to depression treatment: combined psychotherapy and pharmacotherapy was found more effective at treating depression among adolescents with a history of sexual abuse than other treatment plans, while all types of treatment showed similarly effective for adolescents without violence experience.^39^ Our findings demonstrate that adolescents with depression and recent experiences of violence have higher risk of suicidal ideation compared with their counterparts without such encounters. Taken together, these studies consistently highlight the importance of identifying and accounting for violence encounters in depression management, from assessing suicide risk to determining a treatment plan.^40^

The mechanism underlying the excessive risk of suicidal ideation associated with violence encounters among adolescents with depression is not well understood. However, comorbid substance use or posttraumatic stress disorder—2 sequelae of violence encounters—are known to increase risk for suicidal behaviors among patients with depression.^41,42,43,44^ Suffering from violence and associated injury may also decrease one’s fear of death and increase suicidal ideation.^6,45^ Lack of coping skills and cognitive processes following traumatic experiences may also be associated with risk. Maltreated adolescents can exhibit diminished cognitive processes similar to adolescents with suicidal ideation (eg, catastrophizing, overgeneralization, black-and-white thinking, and hopelessness).^6,46,47^ Beyond individual level factors, social and environmental factors also warrant consideration. Family level factors, such as economic stress, impaired parent-child relationship, parental mental illness and substance use, and community level factors, such as concentrated poverty, high violence, and poor neighborhood cohesion are all associated with interpersonal violence.^48,49,50^ In these contexts, lacking social supports, connectedness, and interpersonal difficulties may further exacerbate the risk of suicidal behavior following violence encounters.^51^ To address these multilevel factors, comprehensive primary prevention approaches can be effective in preventing multiple types of violence, including suicidal risk.^52,53,54^

### Limitations

This study had limitations. All conditions were defined via clinical codes, which underestimates the total number of cases as many may not seek care or receive a diagnosis, and misclassification might exist. For example, in our study, 13.7% of adolescents documented suicidal ideation within a year following depression diagnosis, which is lower than the annual rate for self-reported suicidal ideation (ranging from 30.0% to 42.0% for adolescents aged 14–21 years with depression).^4^ Similarly, compared with 1.6% of adolescents with depression who had past-year violent experience in our study, self-reported data from the National Survey of Children’s Exposure to Violence indicated a much higher prevalence of past-year violence among adolescents aged 10 to 17 years (ie, about 40%–46% for physical assault and 17%–21% for maltreatment).^55^ However, only 1.9% made medical visits following violence exposure (yet this is still equivalent to 1.4 million children and adolescents),^31^ which may largely explain the low rate of violence encounters observed in our study. Underreporting of violence encounters, leading to those with encounters being misclassified as without encounters, may bias the association of violence encounters with suicidal ideation toward null. Substance use was also likely underdiagnosed as it may not be disclosed or detected. Inability to fully adjust for substance use may affect our findings. Reassuringly, a prior community-based study found the relative risk of exposure to assault for suicide attempts was minimally changed by adjusting for preexisting substance use.^56^

With a limited follow-up period, we could not define the chronicity of violence encounters. Similarly, we were able to identify the status of depression and suicidal ideation according to diagnoses at a certain point after violence encounters, but those may not be the initial occurrences. There lacks a complete picture of the sequences of the events. Additionally, we could not account for family- and community-level factors in the analyses. Therefore, no causal relationships could be inferred from our study. Given that violence experiences that prompt medical visits are usually severe,^31^ it is unclear whether our results apply to general violence exposure. Population-based studies are warranted to address these limitations. Moreover, if patients received care from practitioners not participating in Explorys, these diagnoses would not be captured in the data. It is unclear whether this issue would vary by violence encounter status. As electronic health records generally have low quality data on race and ethnicity,^57^ often race or ethnicity alone or neither were documented in Explorys. We combined ‘missing’ with ‘other’ for race and ethnicity and insurance type in our analyses, preventing us from making interpretations on this population.

## Conclusions

This study suggests that recent violence encounters are associated with elevated risk for documented suicidal ideation among adolescents with depression. Recognizing recent violence encounters can provide important information for suicide risk assessment and intervention at the time of depression diagnosis. Preventing violence from occurring in the first place, and mitigating the negative impacts when it occurs, has the potential to prevent later excessive morbidity from depression and suicidal ideation.

## Notes

Supplement 1. **eFigure.** Study Design of the Cohort **eTable 1. **Algorithm Used to Define Conditions Included in the Current Study **eTable 2. **Specified Timings Relative to the Index Date in the Definitions of the Main Variables **eTable 3. **Distribution of Race and Ethnicity Among Adolescents Who Were Exposed to Violence in National Population-Based Surveys vs in Explorys **eTable 4. **Distribution in Race and Ethnicity and Insurance Type Among Adolescents With Depression in National Survey on Drug Use and Health (NSDUH) vs in Explorys

Supplement 2.
Data Sharing Statement
