# Supplementary material for: Association of Recent Violence Encounters With Suicidal Ideation Among Adolescents With Depression
Source: JAMA Netw Open. 2023 Mar 2;6(3):e231190. doi: 10.1001/jamanetworkopen.2023.1190 (PMC9982692; doi:10.1001/jamanetworkopen.2023.1190)
Supplement: Supplement 1. — eFigure. Study Design of the Cohort eTable 1. Algorithm Used to Define Conditions Included in the Current Study eTable 2. Specified Timings Relative to the Index Date in the Definitions of the Main Variables eTable 3. Distribution of Race and Ethnicity Among Adolescents Who Were Exposed to Violence in National Population-Based Surveys vs in Explorys eTable 4. Distribution in Race and Ethnicity and Insurance Type Among Adolescents With Depression in National Survey on Drug Use and Health (NSDUH) vs in Explorys [file jamanetwopen-e231190-s001.pdf]

Supplemental Online Content

Wang J, Harrer S, Zwald ML, et al. Association of recent violence encounters with suicidal ideation among adolescents with depression. *JAMA Netw Open*. 2023;6(3):e231190. doi:10.1001/jamanetworkopen.2023.1190

eFigure. Study Design of the Cohort

eTable 1. Algorithm Used to Define Conditions Included in the Current Study

eTable 2. Specified Timings Relative to the Index Date in the Definitions of the Main Variables

eTable 3. Distribution of Race and Ethnicity Among Adolescents Who Were Exposed to Violence in National Population-Based Surveys vs in Explorys

eTable 4. Distribution in Race and Ethnicity and Insurance Type Among Adolescents With Depression in National Survey on Drug Use and Health (NSDUH) vs in Explorys

This supplemental material has been provided by the authors to give readers additional information about their work.

© 2023 Wang J et al. *JAMA Network Open.*

eFigure. Study Design of the Cohort

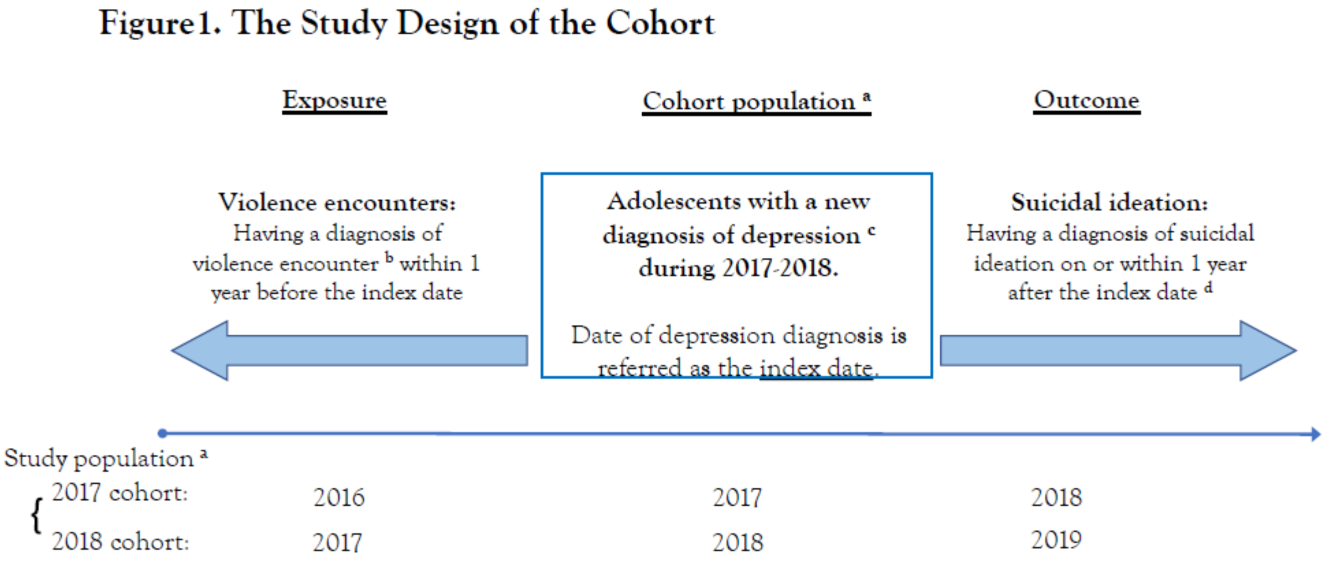

Data source: IBM Explorys Electronic Health Record Database. All conditions were defined by *ICD-10-CM* codes as shown in eTable 1. [a]The final cohort is a combined population of 2017 cohort and 2018 cohort. A 2017 cohort was based on 3-year follow up period from 1/1/2016-12/31/2018. Similarly, a 2018 cohort was based on 1/1/2017-12/31/2019. [b]Violence encounters included child maltreatment (ie, child physical, sexual, or psychological abuse or neglect) or physical assault. [c]A new diagnosis of depression was defined as without documented depression during the 1 year before the index date, which might indicate a new incidence or new episode of depression. [d]Patients who were diagnosed with suicidal ideation before the index date were excluded from the cohort.

© 2023 Wang J et al. *JAMA Network Open.*

eTable 1. Algorithm Used to Define Conditions Included in the Current Study

Condition	Coding system	Codes	Description	Note
**Mental illness or substance use**[a]				
Depression	ICD-10-CM	F32	Major depressive disorder, single episode	
		F33	Major depressive disorder, recurrent	F33 was included to identify depression diagnosis before the index date but was not included to define a new diagnosis of depression.
		F43.21	Adjustment disorder with depressed mood	
		F43.23	Adjustment disorder with mixed anxiety and depressed mood	
Other mental illness	ICD-10-CM	F84	Autism spectrum disorder	
		F90	Attention-deficit hyperactivity disorders	
		F91	Conduct disorders	
		F30	Manic episode	
		F20	Schizophrenia	
Substance use disorder	ICD-10-CM	F10	Alcohol related disorders	
		F11	Opioid related disorders	
		F12	Cannabis related disorders	
		F13	Sedative, hypnotic, or anxiolytic related disorders	

© 2023 Wang J et al. *JAMA Network Open.*

		F14	Cocaine related disorders	
		F15	Other stimulant related disorders	
		F16	Hallucinogen related disorders	
		F17	Nicotine dependence	
		F18	Inhalant related disorders	
		F19	Other psychoactive substance related disorders	
	Patient self-reported behavior	'Alcohol_y'/ 'Alcohol_quit'	Alcohol use	
		'Tobacco_y'/' Tobacco_quit'	Tobacco use	
**Violence encounter**[b]	ICD-10-CM	T74, T76	Child maltreatment	
		X92-Y09	Assault	
**Form of violence**[b]	ICD-10-CM	T74.0, T76.0	Child neglect	
		T74.1. T76.1	Child physical abuse	
		T74.2, T76.2	Child sexual abuse	
		T74.3, T76.3	Child psychological abuse	
		X92-Y09	Assault	
**Suicidal ideation**[b]	ICD-10-CM	R45.851	Suicidal ideation	
**PHQ-9 assessment**	LOINC			
		44261-6	Patient health questionnaire 9 item total score	
		44249-1	PHQ-9 quick depression assessment panel	

© 2023 Wang J et al. *JAMA Network Open.*

		44260-8	Thoughts that you would be better off dead, or of hurting yourself in some way in last 2W	This single item was included to particularly capture evaluation on suicidal ideation.
**Depression treatment**	SNOMED-CT			
Antidepressant prescription		372720008 (and child codes)	Antidepressant	

Abbreviations: ICD-10-CM: International Classification of Diseases, Tenth Revision, Clinical Modification; LOINC: Logical Observation Identifiers Names and Codes; SNOMED CT: Systematized Nomenclature of Medicine Clinical Terms.

[a] The algorithms for mental health illness and substance use were adopted from publicly-available list of codes on mental health used by Mental Health Research Network, a public domain to facilitate mental health research (available at https://github.com/MHResearchNetwork/Diagnosis-Codes).

[b] The algorithms for violence encounter and suicidal ideation were adopted from National Health Statistics Reports: The International Classification of Diseases, 10th Revision, Clinical Modification (ICD–10–CM) external cause-of-injury framework for categorizing mechanism and intent of injury (available at https://www.cdc.gov/nchs/data/nhsr/nhsr136-508.pdf

© 2023 Wang J et al. *JAMA Network Open.*

eTable 2. Specified Timings Relative to the Index Date in the Definitions of the Main Variables

Variable category	Variable name	Case definition	Relationship to the index date (date of depression diagnosis)	
			Before the index date	After the index date
Exposure	Violence encounter	Having a diagnosis of violence encounter within 1 year before the index date	x	
Outcome	Suicidal ideation	Having a diagnosis of suicidal ideation on or within 1 year after the index date		x
Covariates	Mental illness	A diagnosis of mental illness any time prior to the index date	x	
	Substance use	For nonencounter group: a diagnosis of substance use any time prior to the index date	x	
		For encounter group: a diagnosis of substance use any time prior to the latest violence encounter	x	

© 2023 Wang J et al. *JAMA Network Open.*

eTable 3. Distribution of Race and Ethnicity Among Adolescents Who Were Exposed to Violence in National Population-Based Surveys vs in Explorys

**Data source**		**NatSCEV [a]**						**NCANDS [b]**	**Explorys**
Type of violence		Sexual abuse [c] (Age 0-17y in 2008,2011,2014)			Corporal punishment [d] (Age 10-17y in 2014)			Child maltreatment [e] (Age 0-17y in 2017)	Child maltreatment and assault
	Total U.S. population for age group 10-17y in 2017[f]	Rate (per 100 persons) [c]	Number of persons with violence encounters [g]	**%[h]**	Rate (per 100 persons)[d]	Number of persons with violence encounter [g]	**%[h]**	**%[e]**	**%**
Race/ethnicity									
Non-Hispanic White	18016499	3.1	558511	**45.5**	19.00	3423135	**44.6**	**44.6**	**51.6**
Non-Hispanic Black	4996373	5.7	284793	**23.2**	27.00	1349021	**17.6**	**20.7**	**21.4**
Hispanic	8134440	4.1	333512	**27.2**	25.00	2033610	**26.5**	**22.3**	**11.6**
Other	2259702	2.2	49713	**4.1**	N/A	877847.7	**11.4**	**12.4**	**15.3**

© 2023 Wang J et al. *JAMA Network Open.*

[a] The National Survey of Children's Exposure to Violence (NatSCEV) is a periodic survey on nationally representative sample of children and youth ages one month to 17 years of age (details available at National Survey of Children's Exposure to Violence (NatSCEV) | Crimes against Children Research Center (unh.edu)). It is conducted by telephone interviewing on exposures to a wide range of violence.

[b] National Child Abuse and Neglect Data System (NCANDS) is collected annually by Children's Bureau in the Administration on Children, Youth and Families, administration for Children and Families (details available at Child Maltreatment 2017 (hhs.gov)). It contains national number of child maltreatment cases reported to child protective services (CPS) agency.

[c] Gewirtz-Meydan A, Finkelhor D. Sexual Abuse and Assault in a Large National Sample of Children and Adolescents. Child Maltreatment. 2019;25(2):203-214. doi:10.1177/1077559519873975

[d] Finkelhor D, Turner H, Wormuth BK, Vanderminden J, Hamby S. Corporal punishment: Current rates from a national survey. Journal of Child and Family Studies. 2019;28(11) doi:10.1007/s10826-019-01426-4

[e] U.S. Department of Health & Human Services, Administration for Children and Families, Administration on Children, Youth and Families, Children's Bureau (2019) Child Maltreatment 2017. Available from https://www.acf.hhs.gov/cb/research-data-technology/statistics-research/child-maltreatment.

[f] CDC WONDER Online Database at http://wonder.cdc.gov/bridged-race-v2020.html

[g] Calculated by applying the rate (of sexual abuse [c] or corporal punishment [d]) for each race/ethnicity subgroup to corresponding U.S. population number [f].

[h] Distribution in race/ethnicity was calculated as following: we first obtained the total number of persons with violence encounters by summing up the number of persons with violence encounters in each race/ethnicity category (as derived in note [g]). Then, number of persons with violence encounters in each race/ethnicity category was divided by the total number of persons with violence encounters to obtain the distribution of race/ethnicity among persons with violence encounters.

© 2023 Wang J et al. *JAMA Network Open.*

There were slightly higher percentage of White among adolescents with violence encounters in our data compared to other data sources. As described in Methods section, Hispanic ethnicity was not accurately documented in Explorys. Afterall, the comparison does not suggest that non-White minorities were overrepresented in our study.

© 2023 Wang J et al. *JAMA Network Open.*

eTable 4. Distribution in Race and Ethnicity and Insurance Type Among Adolescents With Depression in National Survey on Drug Use and Health (NSDUH) vs in Explorys

	NSDUH[a]		Explorys	
	Had Major Depressive Episode (MDE) in past year		Newly diagnosed with depression	
	Number in thousands	%	Number	%
Race/ethnicity				
Non-Hispanic White	1905	54.7	13437	55.9
Non-Hispanic Black	334	9.6	2354	9.8
Hispanic	882	25.3	1692	7.0
Other	362	10.4	6564	27.3
Insurance type				
Private	2050	56.1	12939	53.8
Public	1249	34.2	5516	22.9
Other/missing	356	9.7	5592	23.3

[a] Among persons aged 12 to 17 years based on NSDUH 2018 data available at 2018 NSDUH Detailed Tables | CBHSQ Data (samhsa.gov). NSDUH is a nationally representative survey of noninstitutionalized civilian aged 12 or older in the United States. The survey, sponsored by the Substance Abuse and Mental Health Services Administration (SAMHSA), is conducted annually via face-to-face household interview on substance use and mental health issues.

The percentages of white and black were similar in Explorys and NSDUH. As described in Methods section, Hispanic ethnicity was not accurately documented in Explorys. The percentage of private insurance was similar in Explorys as in NSDUH. This comparison does not suggest that adolescents of non-white minorities or with non-private insurance were over-represented in our study population.

© 2023 Wang J et al. *JAMA Network Open.*
