# Supplementary material for: Association of Recent Violence Encounters With Suicidal Ideation Among Adolescents With Depression
Source: JAMA Netw Open. 2023 Mar 2;6(3):e231190. doi: 10.1001/jamanetworkopen.2023.1190 (PMC9982692; doi:10.1001/jamanetworkopen.2023.1190)
Supplement: Supplement 2. — Data Sharing Statement [file jamanetwopen-e231190-s002.pdf]

Data Sharing Statement

Wang. Association of Recent Violence Encounters With Suicidal Ideation Among Adolescents With Depression. *JAMA Netw Open*. Published March 02, 2023.
doi:10.1001/jamanetworkopen.2023.1190

Data
Data available: No

Additional Information
Explanation for why data not available: Explorys was owned by IBM Watson Health (now is Merative™). Data was obtained under a data user agreement that explicitly prohibits data sharing. More info on data access can be found at https://www.ibm.com/watson-health/about/explorys
